# Exploring Anorectal Manometry as a Method to Study the Effect of Locally Administered Ropivacaine in Patients with Ulcerative Colitis

**DOI:** 10.1155/2013/656921

**Published:** 2013-02-17

**Authors:** Eva Arlander, Robert Löfberg, Leif Törkvist, Ulrik Lindforss

**Affiliations:** ^1^Division of Clinical Pharmacology, Department of Laboratory Medicine, Karolinska Institutet, Karolinska University Hospital, 141 83 Stockholm, Sweden; ^2^Department of Inflammatory Bowel Diseases, HMQ Sophia Hospital, 114 86 Stockholm, Sweden; ^3^Department of Clinical Science, Innovation and Technology, Karolinska Institutet and IBD Clinical Research Group, Karolinska University Hospital, 141 86 Stockholm, Sweden

## Abstract

The symptoms of distal ulcerative colitis have been related to changes in rectal sensitivity and capacity due to inflammation, altered gastrointestinal motility, and sensory perception. With the use of anorectal manometry, the function was measured in seven patients with active distal proctitis during local treatment with ropivacaine. Seven healthy subjects were studied in the same way for comparison with normal conditions. The anal resting pressure and squeezing pressure were similar in all groups. Significantly lower rectal distention volumes were required for rectal sensation, critical volume, and to induce rectal contractility in patients with active disease compared to controls. Rectal compliance was significantly reduced in patients with active and quiescent disease. The increased rectal sensitivity and contractility in patients with active colitis appear to be related to active mucosal inflammation and ulceration. The frequency and urgency of defecation and the fecal incontinence may be due to a hypersensitive, hyperactive, and poorly compliant rectum. The findings in our study indicate that the inflammatory damage to the rectal wall with poor compliance is unaffected by local anaesthetics such as ropivacaine. The symptomatic relief and reduction in clinical symptoms following treatment are not reflected in the anorectal manometric findings.

## 1. Introduction

Ulcerative colitis (UC) is characterised by intermittent flares of active disease with bowel symptoms. These symptoms include an increased frequency of bowel movements, urgency, sensation of incomplete evacuation, and tenesmi [1]. Alterations in colonic motility may contribute to the increased urgency and frequency of defecation [2, 3], and an increased rectal sensitivity and reactivity is associated with active inflammation [4–7]. Clinical symptoms in active UC are considered to be secondary to the inflammatory process reflecting alterations in the function of smooth muscle, enteric neurotransmission, or afferent sensory input from the bowel wall [8–11].

A long-acting local anaesthetic, ropivacaine, has been explored as a potential therapy for UC [12]. An open clinical study of patients with active distal CU treated with 200 mg rectal ropivacaine gel twice daily indicated a prompt symptomatic relief including a decrease in the number of stools and tenesmi [13]. In addition to reversible block of nerve pulse propagation, ropivacaine affects a variety of cell functions in the inflammatory response [14–18]. Thus the reduction of clinical symptoms can be related to an attenuation of the sensitivity and reactivity of the inflamed rectum resulting from both the anti-inflammatory activity and an action on enteric nerves. Previous animal data indicate that ropivacaine can stimulate colonic contractility, possibly mediated by a direct effect on enteric nerves or smooth muscle cells [19]. Due to a fast onset of nerve block in relation to tissue healing, our hypothesis was that a restoration of rectal function would be seen early on in treatment, before mucosal healing. The aim of our study was to explore a method to follow this symptomatic effect in combination with traditional methods including endoscopy and assessment of clinical symptoms. Anorectal manometry has been shown to capture rectal sensitivity, capacity, and reactivity [20, 21]. The method has previously been demonstrated to distinguish between active and quiescent UC [4–6] and has also been tested as a biomarker for irritable bowel syndrome [22]. To our knowledge, this is the first study to utilize anorectal manometry to study a clinical effect over a treatment period.

Rectal sensitivity threshold, capacity, and rectal pressures were exploratively studied in seven patients with distally located UC with a comparison to seven healthy subjects. Manometry investigations in the patient group were performed before and during four weeks of daily rectal therapy with 150 mg ropivacaine gel.

## 2. Materials and Methods

### 2.1. Patients

Patients were eligible for the study if they were between 18 and 70 years old and diagnosed with mild-to-moderate distal UC characterized by colonoscopy at inclusion (grade 2 or higher; see Table 1). The patients should have had rectal bleeding at least once during the last week prior to study. Patients on maintenance therapy with oral 5-ASA derivates were allowed to continue treatment. Exclusion criteria included (1) rectal therapy within three days of the start of study treatment, (2) rectal lidocaine gel within two weeks of the start of study treatment, (3) pregnancy or lactation, (4) sensitivity to local anaesthetics of the amide type and (5) clinically significant hepatic, renal, cardiovascular, or other concomitant diseases that required drug therapy. During the study, treatment with any other local anaesthetic was not allowed, neither was any other rectal medication. Medications that could affect the bowel motility, for example, purgatives and anticholinergic or antidepressant drugs, were not allowed during the study.

Seven patients with UC (5 males) between the ages of 20 and 54 (median age 27 years) were included. All had a confirmed diagnosis of mild-to-moderate active distal UC. The median disease duration since first onset of UC was 5 years. Time from last exacerbation was 3 years. Oral maintenance therapy was continued in three patients: mesalazine 3 g daily and sulphasalazine 4.4 and 3 g, respectively.

### 2.2. Controls

Seven healthy subjects (5 males) between the ages of 19 and 50 (median age 37 years) were recruited. None of the subjects had any evidence of anorectal dysfunction or diseases interfering with the control of the small and large intestines.

All patients and healthy subjects gave their written informed consent before entering the study, which was done in accordance with the Principles of the Declaration of Helsinki and was approved by the Local Human Ethics Committee at Karolinska Institutet, Huddinge University Hospital, Stockholm, Sweden.

### 2.3. Study Drug and Study Design

Ropivacaine hydrochloride, 150 mg, in 20 mL methylcellulose gel, unbuffered with pH 4.4 (Astra Pain Control AB, Sweden) was administered rectally twice daily during four weeks.

Compliance with the treatment was checked by interview and count of medication at each clinic visit. The total dose for complete compliance during study was 8400 mg. The patients that completed the study according to the protocol had taken a total dose between 7350 and 8400 mg, and four patients had missed between one and three doses. The patients were scheduled for clinic visits including clinical assessments and anorectal manometry investigations before study start, after two days, one week, and four weeks of treatment.

### 2.4. Endoscopic and Histological Assessments

Flexible video colonoscopy or a rigid sigmoidoscope was used, depending on the extent of disease, to determine the proximal extent and to assess the severity of the inflammatory changes before treatment start (baseline) and after two days and one and four weeks of treatment. The appearance of the mucosa at the most severely affected site was rated on a scale from 0 to 3 (Table 1). The distance from the anal verge to healthy tissue was recorded at baseline and at week four.

Biopsies were taken at baseline and after one and four weeks of treatment. One biopsy was sampled from the most inflamed area of colorectum at each time point. In addition, one biopsy from healthy tissue proximal to the inflamed area was taken at baseline. Biopsies were evaluated in a blinded manner by an independent histopathologist according to the grading scale for histological assessment of inflammation in ulcerative colitis described by Geboes et al. [23].

### 2.5. Clinical Assessments

Patients recorded bowel habits and presence of diarrhea, mucus, urgency, pain, and blood as the average of the last three days before each visit at baseline and after two days and one and four weeks of treatment. Blood tests and urine analysis were taken at baseline and after one and four weeks of treatment. 

### 2.6. Treatment Effect

The treatment effect was expressed in terms of remission and improvement. Remission was defined as an endoscopic score <1, rectal bleeding = 0, and either rectal or abdominal pain = 0.

Improvement was defined as a decrease in the total score for disease activity by more than 3 points compared to baseline.

### 2.7. Anorectal Manometry

We used on open tip perfused (0.4 mL/min) catheter system, comprising an eight channel polyvinyl catheter (length 90 cm, outer diameter 4.8 mm), a pneumohydraulic infusion pump (Andorfer Medical Specialities, Greendale, WI, USA), and a computerized data monitoring system (polygraph 12 HR, Synthetics Medical, Stockholm, Sweden). The pressure recording points were located at the same level, separated by an angle of 45°. Resting and squeezing pressures were measured by a continuous pull-through (speed 2.5 mm/s) by means of a withdrawal system. The resting and squeezing rectoanal pressures were calculated from the mean of five (resting) and three (squeezing) measurements.

Rectal sensitivity threshold and capacity and the rectoanal inhibitory reflex (RAIR) were studied by using a 4-lumen catheter with a latex balloon (5 × 5 cm) at the tip. The balloon was continuously filled with water at a speed of 4 mL/min. Three parameters of sensitivity were measured: first sensation (conscious rectal sensitivity), urge threshold (constant urge to defecate), and maximum tolerable volume. This implies that the patient's own tolerance towards different volumes during a defined period of time was measured. Cut-off volume was at 250 mL. RAIR was defined as the lowest balloon volume required eliciting a sustained relaxation of the internal anal sphincter. No bowel preparation was used for the measurements, but the patients/subjects had to evacuate the bowel before the examination. All recordings were done with the patient/subject in the right lateral position.

### 2.8. Statistical Evaluation

Results are presented as medians (min/max). Individual data have been emphasized in this explorative study. Analysis of variance (ANOVA) was used to compare healthy subjects and patients with respect to manometry data. The level of significance was two-sided and 5%.

## 3. Results

### 3.1. Clinical Effect

Two patients were withdrawn from the study due to adverse events. One patient had a deterioration of UC that led to hospitalization after 26 days of treatment. The second patient had an allergic reaction (angioedema) on treatment day 17 and urgent medical treatment. This patient had attained clinical and endoscopic remission at the time of the event.

**Figure 1:**
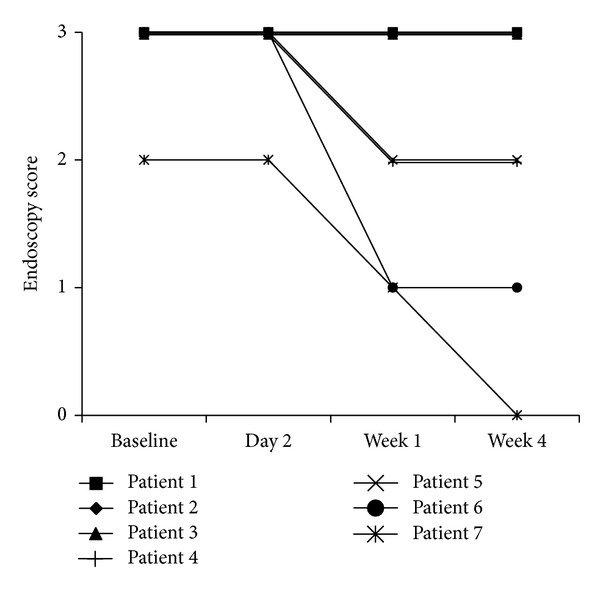
Endoscopy scores before start of treatment (baseline) and after two days and one and four weeks of treatment with 150 mg ropivacaine gel twice daily in patients with distal UC.

**Figure 2:**
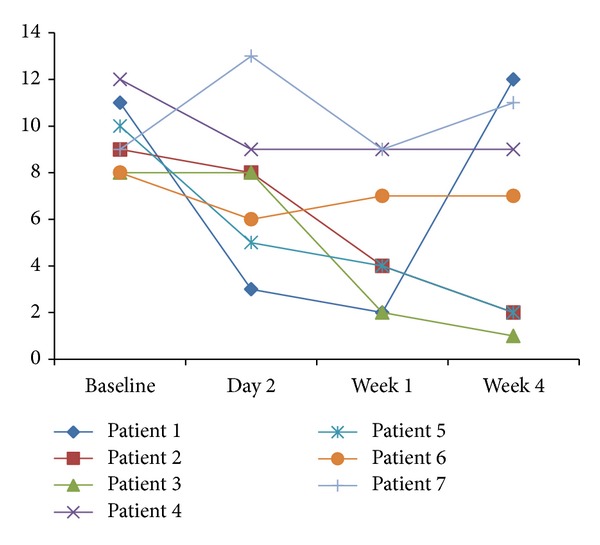
Summary of clinical symptoms before start of treatment (baseline) and after two days and one and four weeks of treatment with 150 mg ropivacaine gel twice daily in patients with distal UC.

The endoscopic and histological scores decreased as well as stool frequency, urgency, and rectal bleeding (Figures 1 and 2 and Table 2). Clinical improvement was seen early, and most patients improved during the first week of treatment. Two patients (no. 2 and no. 3) attained remission. Two patients improved (no. 4 and no. 5).

### 3.2. Anorectal Manometry

**Figure 3:**
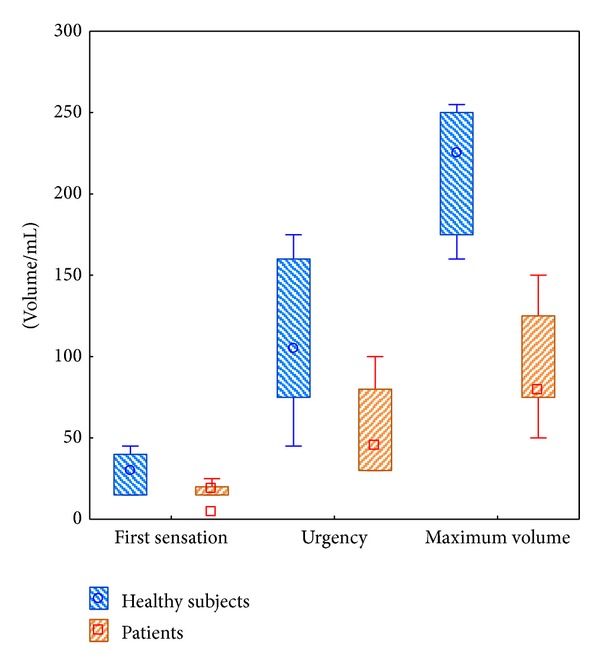
The volumes required to elicit the first sensation, the sensation of urgency, and the maximum filling volume before the start of treatment in patients with distal UC (*n* = 7) compared with healthy subjects (*n* = 7).

Overall, rectal sensitivity showed a larger variability between individuals than within. The volumes that induced first sensation were similar (NS) between patients and controls, while the differences in volumes to induce urgency (*P* < 0.05) and maximum tolerance (*P* < 0.001) were 2- to 3-fold higher in healthy subjects (Figure 3 and Table 3).

On average, threshold volume for first sensation, sensation of urgency, and maximum filling was unchanged in the patient group. On an individual basis, patient no. 2 had a slightly increased tolerability to rectal distension. This patient and patient no. 3 attained clinical and endoscopic remission. In patient no. 3, however, no increase in volume tolerability was noted.

In all patients the rectoanal inhibitory reflex (RAIR) was present, and the volumes to induce RAIR did not change during the treatment period.

Anorectal pressures at rest and on squeezing were similar over the treatment period among patients, but in comparison with healthy controls patients had higher rectal pressures, *P* < 0.05, respectively (Table 4).

### 3.3. Tolerability

Adverse events were recorded from the start of treatment until two weeks after the last dose. Reported events relating to UC included abdominal pain, conjunctivitis, fatigue, and constipation (all reported once, resp.). Other symptoms were paraesthesia in hands and feet, sweating, back and chest pain, eczema, and vertigo. Two patients were withdrawn from the study due to adverse reactions (see aforementioned). The patient with the angioedema was treated with rectal lidocaine gel when the allergic reaction had ceased. No new symptoms developed. No clinically relevant changes were seen in the laboratory examinations, electrocardiograms, pulse, or blood pressure in any of the patients. None of the healthy subjects reported any adverse effects in connection with the anorectal manometry investigations.

## 4. Discussion

The aim of our study was to explore if anorectal manometry could be suitable as a method to describe and quantify the pharmacodynamic effects during rectal treatment with the local anaesthetic ropivacaine.

The overall rectal sensitivity and tolerability to distension were unchanged during the four-week treatment, despite an improvement in clinical symptoms and mucosal healing.

All patients recruited to our open study had active distal mucosal inflammation, that is, proctitis or proctosigmoiditis including symptoms of rectal bleeding, urgency, and diarrhea. In comparison to the controls, they had an increased sensitivity and reactivity towards rectal distension at study start. The prospective follow-up of rectal sensitivity threshold and capacity during ropivacaine treatment provided good opportunities to discover a relationship between these recordings and treatment effects. However, the overall improvement in clinical symptoms and endoscopic scores, with two patients attaining full remission, was not parallel to changes in anorectal assessments.

Symptoms of bowel discomfort as experienced by UC patients during disease exacerbation are partially related to the greater degree of inflammation present during flares resulting in transient sensitization of afferent pathways [5, 6, 24]. The hypothesis was that application of ropivacaine would attenuate rectal hypersensitivity by action on enteric nerves and lead to an improvement in symptoms like urgency and frequent stools. Furthermore, Martinsson et al. [19] showed restoration of contractile activity in colonic muscle strips after treatment with ropivacaine in rats with trinitrobenzeene-induced colitis. This effect was seen before mucosal healing and suggested either a direct effect of ropivacaine on smooth muscle cells or an anti-inflammatory effect leading to an increased muscle force or an indirect effect mediated by inhibition of tonic inhibitory afferent signals. Thus, the restoration of colonic motility by ropivacaine may explain a symptomatic relief occurring prior to mucosal healing. Some patients in our study experienced an improvement in the bowel symptoms, but these were not accompanied by changes in rectal sensitivity threshold and capacity or rectal pressures.

The increased sensitivity of rectum has also been related to the presence of inflammation [2, 4, 5, 7, 25, 26]. Accordingly an anti-inflammatory action could be beneficial resulting in restoration of anorectal sensitivity and tolerability to distension. Only one of the two patients in our study that experienced remission with mucosal healing and symptomatic improvement had a tendency to a decreased rectal sensitivity and reactivity.

There are some discrepancies with respect to the methods of performance for the anorectal manometry tests [20, 27], although standardization is under way [28]. This implies that any comparisons with previously published data must be made with care since factors like size and placement of the balloon, flow rate, and stationary or pull-through technique may differ and influence results. It is therefore important to include controls in studies. Previously, patients with colitis have been studied with respect to rectal sensitivity and function with methods similar to the present study. Previous results showed that there was a 1.5- to 4-fold difference in the volume required to elicit first sensation and urgency between colitis patients and healthy subjects or patients with inactive disease [4, 5, 29]. The first sensation to intrarectal balloon distension occurred at mean volumes between 13 and 21 mL in patients with active disease. For sensation of urgency and maximum tolerable volume corresponding values were between 35 and 40 mL and 80 and 100 mL, respectively. Thus our data are in line with previously recorded data, both in patients and controls, and verify that patients with active colitis have an increased sensitivity and reactivity of rectum.

The ropivacaine dose (150 mg daily) in this study was 50 mg lower than the dose in our first study in patients [13]. The treatment period was fairly short and may have affected the possibilities to record changes in the anorectal function, sensitivity, and tolerability to distension. 

This study is explorative, a fact that gives limitations to the conclusions that can be made. Even though the number of patients was small, our results showed good reproducibility over time within individuals. The findings in our study indicate that the inflammatory damage to the rectal wall with thickening and impaired capacity, including poor compliance, is unaffected by local anaesthetics such as ropivacaine. The higher resting pressure among UC patients is a probable, compensatory mechanism. Still there is symptomatic relief and reduction in clinical symptoms following treatment, and these are not reflected in the anorectal manometric findings.

## Figures and Tables

**Table 1:** Endoscopy scale.

	Endoscopy grading scores
0	Noninflamed mucosa
1	Granularity, oedema, lack of normal vascular pattern
2	Hyperaemia, friability, petechiae (and all of score 1)
3	Ulceration (and all of scores 1 and 2)

**Table 2:** Clinical symptoms, endoscopy, and histology scores as recorded before start of treatment (baseline) and after two days and one and four weeks of treatment with 150 mg ropivacaine gel twice daily for four weeks in patients with distal UC.

Time (*n* = 7)	Rectal bleeding		Rectal pain		Stool consistency		Urgency		Summary of clinical symptoms		Endoscopy		Histology	
	Median	Min/max	Median	Min/max	Median	Min/max	Median	Min/max	Median	Min/max	Median	Min/max	Median	Min/max
Baseline	2.0	2.0/2.0	1.0	0.0/2.0	1.0	1.0/2.0	3.0	2.0/3.0	9.0	8.0/10.0	3.0	2.0/3.0	5.0	5.0/5.0
Day 2	2.0	1.0/2.0	1.0	1.0/1.0	1.0	1.0/2.0	2.0	1.0/3.0	8.0	6.0/9.0	3.0	2.0/3.0	NP	
Week 1*	1.5	0.5/1.5	1.5	0.5/2.0	0.5	0.5/2.0	1.0	1.0/2.0	8.0	5.0/9.0	3.0	1.0/3.0	4.5	3.5/5.0
Week 4	1.0	1.0/1.0	1.0	0.0/1.0	1.0	1.0/1.0	1.0	0.0/3.0	7.0	2.0/9.0	2.0	0.0/3.0	3.0	2.0/4.0*

*n* = 6, NP: not performed.

**Table 3:** The volumes required to elicit first sensation, sensation of urgency, maximum filling, and rectoanal inhibitory reflex (RAIR) before start of treatment (baseline) and after two days and one and four weeks of treatment with 150 mg ropivacaine gel twice daily for four weeks in patients with distal UC.

Time (n = 7)	Initial sensation		Initial sensation		Sensation urgency		Sensation urgency		Max tolerable		Max tolerable volume		RAIR	(mL)
	(mL)		controls (mL)		(mL)		(mL)		volume (mL)		(mL) controls			
	Median	Min/max	Median	Min/max	Median	Min/max	Median	Min/max	Median	Min/max	Median	Min/max
Baseline	21	9/39	20	7/27	37	30/82	105	29/176	83	72/122	219	156/219	30	30/45
Day 2	20	8/31			43	29/65			59	46/133			30	20/40
Week 1*	18	11/24			48	26/91			68	49/121			30	25/80
Week 4	21	14/33			48	45/52			77	66/96			34	24/40

*n = 6.

**Table 4:** Resting pressure and squeeze pressure recorded before start of treatment (baseline) and after two days and one and four weeks of treatments with 150 mg ropivacaine gel for four weeks in patients with distal UC.

Time (n = 7)	Resting pressure		Squeeze pressure		Resting pressure		Squeeze pressure controls	
(mmHg)	Median	Min/max	Median	Min/max	Median	Min/max	Median	Min/max
Baseline	113	91/128	198	143/208	63	28/79	92	49/158
Day 2	110	92/127	198	142/206				
Week 1*	112	105/133	170	133/215				
Week 4	108	96/137	174	131/195				

*n = 6.
